# Prepare the patient for future challenges when facing hemodialysis: nurses' experiences

**DOI:** 10.3402/qhw.v9.22952

**Published:** 2014-04-08

**Authors:** Anna Sturesson, Kristina Ziegert

**Affiliations:** School of Social and Health Sciences, Halmstad University, Halmstad, Sweden

**Keywords:** Qualitative content analysis, support, transition, hemodialysis, chronic kidney disease

## Abstract

Chronic kidney disease is a major health problem due to the significant financial burden for the healthcare system and likewise for the patient who needs the treatment. The patient's whole life situation is turned upside down with chronic kidney disease when they are confronted with the forced change to start treatment with hemodialysis. Patients with chronic kidney disease experience a lack of adequate emotional support from nurses during the transition to hemodialysis. The purpose of this study was to explore nurses’ experiences of giving support to patients during the transition to hospital-bound hemodialysis. The study had a qualitative descriptive design with a content analysis approach; eight nurses from four hospitals in the south of Sweden participated. The results showed that the nurses gave threshold support with an openness and awareness of the patient's individual needs during the transition, except that there seemed to be a lack of knowledge and ability to provide emotional support. Patient support during the transition could therefore be absent. Education, at local and national levels, is needed for the nurse to be able to give professional emotional support. Further research is also desired in order to provide nurses with the tools they need to give emotional support, which is of utmost importance.

Support in caring for patients with chronic renal failure (chronic kidney disease [CKD]) poses a challenge for nurses. Assistance should be designed with the intention that the patient must maintain as high a quality of everyday life as possible, while taking into account the medical data (Thorne, Harris, Mahoney, Con, & McGuinness, 2003). A variety of stressors affecting patients in this stage can result in patients suffering from anxiety and depression (Harwood, Locking-Cusolito, Spittal, Wilson, & White, 2005; Thorne et al., 2003). The actual transition from treatment for CKD to hemodialysis (HD) and the consequences of the new situation mean that patients’ lives really are turned upside down by a forced change; they must create a new reality (Kralik, Visentin, & van Loon, 2006). Several studies indicate a lack of adequate support from the nurse while the patient is experiencing the preparation to HD. Honest answers and explanations to questions about disease and treatment are handled in the best possible way as demanded by the patients (Mitchell, Farrand, James, Purtell, & Wyatt, 2009). Also in other areas, such as oncology and cardiology nursing, the patients and their families experienced a lack of nursing support (Feldman-Stewart, Brundage, & Tishelman, 2005; Stewart, Davison, Meade, & Makrides, 2000).

Care of the patients takes place in a renal setting and is provided by nephrologists, nurses, a physiotherapist, a counselor, and a dietician. The goal is to, by various measures, delay the patient's dialysis start. Delaying the patient's dialysis start is done by slowing down the disease and keeping it under control with medical care. Concerning the nursing care, the delay is affected by the nurse's ability to provide support to the patient through informing, giving advice, and giving guidelines according to the nurse's knowledge and experience (Crowley, 2003). The nurse's knowledge and understanding of the patient's individual perception and management of the transition to HD are important for the nurse in order to provide the support that patients need (Molzahn, Bruce, & Shields, 2008). Informational support in the form of communication between nurses and patients is, according to Hagren, Pettersen, Severinsson, Lutzen, and Clyne (2004), flawed, with the result that the patients in their current position of dependence on the nurses feel vulnerable. In the transition to HD, it is therefore important that the nurse sees the patient's individual needs and provides support based on different aspects of nursing care. Klang, Björvell, and Clyne (1999) argue that the transition to HD is less stressful for the patient when the nursing care support is adapted to individual patient needs.

## The importance of support

Support is a central concept in nursing and can be described both as an interaction between nurse and patient, and as an action of nurses (Stewart, Davison, Meade, Hirth, & Weld-Viscount, 2001; Ziegert, Fridlund, & Lidell, 2007). Stewart et al. (2001) define support as an interaction between the patient and the natural network of family and friends, and the nursing profession. Ziegert et al. (2007) define support as an action by the nurse to enable the patient to accept their situation through encouragement and recommendation. The interaction that Stewart et al. (2001) describe takes place through the communication of information, emotional alliance, practical help, and affirmation. Stewart divides the support into information support, affirmation support, emotional support, and instrumental support (Stewart et al., 2000, 2001, 2006)****. In this study, we decided to focus on informational, emotional, and practical support.

Ziegert et al. (2007) point to the importance of the nurse's sincere and genuine interest in the patient as an individual when she provides support to the patient. The nurse affects the quality of the support when she shows an interest in the patients through exhibiting openness during the meeting, which creates opportunities for them to participate as well as for her to familiarize herself with the patients’ entire lives. Stewart et al. (2001) point out that intimate relationships are powerful indicators of support for patients. Furthermore, she says that when the patient experiences a sense of continuity in the situation, the support becomes a coping resource with direct and indirect effects on the patient's medical condition. The job description for nurses working in renal clinics also points out a patient-supporting role in nursing care (Svensk njurmedicinsk sjuksköterskeförening [SNSF], 2009). The first step assesses the patient's quality of life, self-care skills, and knowledge requirements. Furthermore, the nurse needs to plan, implement, and evaluate patient education; give advice and support; and discuss treatment options. In order for the nurse to become more aware of the correct treatment, the patient has to be more active and participate in an open dialogue (SNSF, 2009).

## Transition—process and interactions

Chick and Meleis (2007) argue that the concept of transition is a concern for nurses in the care environment. The word comes from the Latin verb *transpire*, and means to go across or pass over time. Transition has to do with change and can be explained as a proexplainedcess that involves a passage or movement from one state, condition, or location to another (Schumacher & Meleis, 1994). According to Kralik et al. (2006), you cannot equate transition with change. The meaning is deeper than that and can be explained as a psychological process in which patients adapt to a changing reality. Transition is a complex concept that includes process, time span, and perception. The process involves different phases and sequences. The time span indicates an ongoing but bound phenomenon, and the perception has to do with the person's experience of the meaning of the transition. Transition is thus both the process and outcome of complex interactions between the individual and the environment. The concept is change, but all change does not necessarily mean a transition. Transition can be positive or negative, self-selected or non-self-selected, and can include multiple transitions simultaneously over a period of time (Chick & Meleis, 2007; Schumacher & Meleis, 1994).

A prerequisite for the transition is that the patient is aware of the change taking place (Kralik et al., 2006). The nurse can facilitate the patient's awareness of the transition by ensuring that the patient receives the education needed. Mitchell et al. (2009) confirm this by explaining that, in the informational support, the nurse has the opportunity to ensure that the patient has received sufficient education and instruction to be well prepared. When the patient is aware of and understands his situation, his ability to engage increases (Kralik et al., 2006). The nurse notices the involvement of the patient from the patient asking their own questions, asking for support, identifying new ways and means to live and act, adapting previous activities, and trying to give meaning to the facts. A successful transition has taken place when the feelings of anxiety and distress are replaced with a sense of well-being and a sense of having mastered the changed situation (Schumacher & Meleis, 1994). The actual transition may be regarded as completed when the patient feels stable and secure (Chick & Meleis, 2007). The nurse's understanding of the transition process and the patient's experience of it make the nurse more capable of identifying individual needs and providing the relevant support (Fex, Flensner, Ek, & Söderhamn, 2011; Kralik et al., 2006).

Research has been carried out concerning nurses’ experiences of their work environment, burnout, and organization (Flynn, Thomas-Hawkins, & Clarke, 2009; Gardner, Thomas-Hawkins, Fogg, & Latham, 2007). There is a gap in research concerning nurses’ experiences in providing support to the patient during the transition to HD, and more knowledge is needed in this area. The aim of this study was to explore support to the patient in his or her transition from treatment for CKD in a kidney setting to hospital-bound HD, from the nurses’ experiences.

## Methods

The study was a qualitative descriptive design with a content analysis approach (Elo & Kyngäs, 2008; Tong, Sainsbury, & Craig, 2007). Qualitative content analysis focuses on the interpretation of various texts in which interpretation can take place at different levels (Polit & Beck 2008). It is used particularly in behavioral sciences, human sciences, and health sciences. Qualitative content analysis is suitable to use when analyzing the multifaceted and sensitive phenomena characteristic of nursing (Elo & Kyngäs, 2008; Malterud, 2001; Tong et al., 2007).

### Participants

In order to explore the nurse's support to the patient during the transition to HD, selection has been directed at four hospitals in the south of Sweden. Eight renal nurses participated in the study. The four clinics had a total of 241 patients with CKD registered in November 2010, according to the Swedish Renal Registry (SNR). Two nurses from each participating hospital took part. One of the informants was also working at the time as a unit manager, and the other nurses had combined duties at the renal clinic and dialysis clinic or had previously worked with dialysis. The selection was made to obtain a geographical distribution as the total number of existing informants was low. The number of employed nurses per renal setting varied between one and four. All nurses in the study were women as there were no men on duty. The age span ranged from 40 to 51. The professional experience of the nurses ranged from 16 to 27 years. The number of years of experience within renal care, dialysis clinic, and renal clinic varied from 15 to 25 years. One nurse had 3 years of undergraduate education, and the remainder had 2 years. No nurse was trained at an advanced level. One nurse was trained at the bachelor level. Two nurses had had further education within intensive care and oncology. Seven out of eight had additional training in renal care corresponding to half a semester of full-time studies. One of these also reported she had education in kidney transplantation. None of the interviewed informants declined participation in the study.

### Data collection

A pilot interview was conducted with a colleague in order to gain experience in interview techniques and improve the formulated questions. Data collection was conducted using semistructured interviews with eight renal nurses at four hospitals in the south of Sweden during March–May 2010. All hospitals were located in different regions in Sweden. The interviews were conducted by coauthor AS and were recorded with the consent of the informants. The length of the interviews ranged from 30 to 50 minutes. The interview was based on the theme of informative support, emotional support, and practical support. The informants were aware of this issue, and the interview began with the opening question “How do you, together with the patient, plan the nursing care prior to the transition to hemodialysis?” Supplementary questions could be “What do you think when you say …?” Or “Can you describe the situation a little more closely?” Following the interviews, there were opportunities to ask questions, and the informants were encouraged to contact the interviewer if they had any queries.

### Data analysis

Qualitative content analysis was chosen as the analytical method according to the model by Elo and Kyngäs (2008). The analysis followed the three phases: the preparation phase, the organization phase, and the reporting phase. In the first phase, the interviews were listened to and then transcribed. Units of meaning related to the purpose of the study were highlighted in order to obtain an understanding of the data and their whole. The next phase was an open coding in which the units of meaning were condensed, abstracted, and named with a code. These codes were numbered and grouped according to their similarities and differences. Code groups were discussed with coauthor KZ and proved to be consistent between authors. In the next step, the code groups were categorized into 10 subcategories and three main categories. In order to meet the purpose of the study, the names of the categories were changed a second time as they were not focused on the nurse's support for the patient but rather on her own qualities. Subcategories that were linked were put together and were eight in number. Categories were gradually abstracted throughout the analysis process. In order to gain balance in the levels of abstraction of the categories, these were changed again, producing the final result. In the final step of analysis, the study was abstracted to a latent level with the theme “Prepare the patient with threshold support for future challenges when facing HD.” In the last phase of the analysis, a table of categories, main categories, and themes was constructed (Table I), and the result was written down (Elo & Kyngäs, 2008; Malterud, 2001; Tong et al., 2007).

**Table I:** Category table.

Category	Main category	Theme
Focus on the patient as an individual	The essential part of gaining knowledge about the patient	Prepare the patient with threshold support for future challenges when facing hemodialysis
Coaching in critical situations
Preparing in time
Balancing an emotional journey	To learn about the patient with self-awareness in mind	
Building teamwork with the patient	
Practice gave the patient valuable experiences	To strengthen the patient for future challenges	
Learning from others	
Using the closest resources at hand	

## Ethical consideration

The study was approved by the local ethics committee at the University of Halmstad: 90-2010-437. Directors of kidney and dialysis clinics at four hospitals in southern Sweden were contacted: first by telephone and e-mail, then with a letter containing information and the request for approval of the study, which was signed and sent back. In the following step, the unit managers of the four dialysis units were contacted, both by phone and by e-mail. They in turn chose two nurses, both of whom were then contacted by the interviewer (AS) via e-mail and by telephone. During the phone call, time and place were booked for the interview and the informants were given the opportunity to ask questions. One interview was conducted in the nurse's home, and the others were conducted in the nurses’ workplaces in accordance with their own requests. The confidentiality of the study was reinforced prior to the interview. Information was given regarding the informant's voluntary participation and the right to discontinue participation at any time, and an agreement regarding participation in the study was signed.

## Result

The first main category explored the support based on the encounter between nurse and patient in the renal setting. The support given by the nurse focused on meeting the patient by creating trust regarding the patient's everyday life. When the nurse got to know the patient, the support was provided in the form of guidance during decision making and different situations where the patient had to make choices. Time was a major factor that influenced the nurse's ability to provide individual support.

The second main category explored support for the patient based on how the nurse responded to patient needs. An existing and an absence of self-awareness as well as a balance and imbalance in the relationship to the patient were described in this section.

The third main category explores the support from the nurse by using fellow patients, colleagues, and relatives in order to strengthen the patient's new lifestyle. The nurse gave support to the patient by allowing him to meet other patients. Support to the patient was also given by colleagues of the nurse. She was, however, the healthcare professional of the renal team with a responsibility to convey these contacts. Next of kin were seen as important resources for support measures for both the patient and the nurse.

### The essential part of gaining knowledge about the patient

In the first main category, the nurse *focused on the patient as an individual*. Support to the patient in the renal setting began with the nurse getting to know the patient. Great emphasis was placed on the relationship during the regular visits to the renal setting, which in some cases could last for anything from several months to years during kidney disease. The meetings were marked by peace and quiet in an open-minded atmosphere in which the patient would be in focus, feel secure, and have the confidence to talk. As they were getting to know each other and talked, the nurse was able to get an idea about the patient's position in his life and of his experiences of renal care so that she could proceed with the required support. The first priority in a supportive conversation was the relationship between the nurse and the patient as well as the delay of the start of dialysis. One nurse described it as:

I believe that nurses should not talk about hemodialysis the very first time you meet, rather take time to get to know each other and discuss prevention before you discuss treatment.

As much as she was able to, the nurse showed an interest in the individual's needs, interest, and willingness by adapting the informative and practical support and visit intervals at the clinic according to the patient's wishes. Here also, she interpreted the patient's wishes in order to determine the level of information. The information was given gradually, and the amount of information was adapted to the patient's needs. The goal was to be attentive to the individual.

But as I said, we have prepared a memo that we should adhere to, but again it's very individual, both the order of and how much information you bring up at each meeting, and some you need to see a lot more than others. But the ultimate goal is that, in the end, all patients receive similar information, but in slightly different ways.

The informational support of the patient was described as an ongoing information process where planning, continuity, anticipation, and feedback were essential elements. For those parts, the conversation was structured. Furthermore, the conversation was described as a communicative exchange in which the patient's views were important. The design of the support required that the nurse was there for the patient, put a lot of effort into her task, and was flexible in the meeting itself. The informational support was prepared with an overview and preferably with an assurance that the patient had understood the information. Information in the form of brochures, videotapes, and training materials was used. The verbal information was thus reinforced by written information. The patient's medical investigation was in focus, and test results were often the topic of conversation. In order to follow the course of the disease, the creatinine level was an important parameter for patients. Also, the patient's vascular access, which was described as the patient's lifeline, was given a lot of focus in the meetings. The informational support varied between patients depending on how the nurse interpreted each patient as she provided the information. The support received also varied depending on the patient's ability to take in the information. The nurse showed much confidence in her informative role, based on her extensive experience and expertise in the area. One experience the nurse had was that patients sometimes lacked trust in the healthcare sector. Therefore, to be highly accessible at the renal clinic was part of the support. The patient could easily contact the nurse and did not have to wait long, neither for booked appointments nor telephone contact.

And we have very high accessibility also at our clinic, no touchtone phone …. I get a note given to me and if I cannot take the call right away, I call back later the same day, I do not leave it for several days and that makes you feel more secure.

Another way to provide support to the patient was to find solutions and change the negative images. Through positive thinking in the meetings, the nurse supported the patient by observing blockages and updating previous information. The negative experiences that the patient had had through previous contact with the healthcare system would change into something positive. It was important to elicit the patient's concerns and to be involved in the patient's feelings regarding the information. In the meetings with the patient, the nurse's task was to provide support by conveying hope in despair and communicating hope of freedom in the restrictions. Support in day-to-day life was also given at the meetings as the nurse took into account, and facilitated, the day-to-day life of the patient by planning the appointments accordingly. The support proved useful in minimizing restrictions, coordinating, and caring for the patient. Working in small clinics and reorganizing made it easier for nurses to provide practical support in the patient's everyday life while conducting regularly meetings during the treatment. If this is managed as stated above, the social status of the patient will guide the nurse to an improved treatment using a holistic approach. A nurse expresses positive thinking as: “And so I try to form a picture of what is the best, what is the most positive thing at the moment.” The nurse described her support to the patient as *coaching in critical situations*.

Guidance regarding choice of treatment meant providing ample and comprehensive information about various treatment options in accordance with the patients’ opportunities and aspirations. The patient's choice of treatment would be an informed choice and be made with as much freedom of choice as possible. If a treatment option was not possible, the nurse should clearly explain this. The decision to commence treatment and the choice of treatment should be strictly voluntary. The patients who decided to abstain from treatment were also given support. The patient should not regret their treatment of choice. A nurse describes:

Then I talk about both peritoneal dialysis (pd) and blood dialysis (bd) and we talk about bd on several occasions and then I can see a little bit what they are most interested in and get a sense of where they're at. Although I can see that in the beginning they want bd, I know that I will still give them information about pd so that they know what it is about.

One way to provide support to those patients who found it difficult to make decisions regarding treatment was that the nurse made sure and received confirmation that the patient had clearly understood the information. Difficulty in support could occur when the nurse knew what treatment was the most appropriate medically, but the patient or relatives did not understand this, or chose to go against the nurse's opinion. She then chose to accept the situation without feelings of failure or professional defeat. To provide support through guidance was not the same as forcing someone to make the “right” decision, and it was described by two nurses like this:

And it's not like I'm going to force him, as he is so firm in his belief. All I can do is to provide the information about dialysis.

It's more like we discuss a bit with the doctor first to see if this is a patient that could have pd. Then we try to push a bit more for pd, but it's not like we are trying to force anyone.

Time was very important for the nurse when she gained knowledge about the patient. Her goal in the support was to *prepare the patient for HD in time*.

A common experience among the nurses was that the patient experienced the transition to be less stressful when there was plenty of time for meeting, gathering information, and planning the transition. Timescale had a prominent role in the nurse's support to the patient during the transition to HD. The nurse's experience was that a long information process was optimal. When there was plenty of time, the information could more easily be adapted to the individual, being general in the beginning and more specific at the end. One nurse described:

It was not the long process, with frequent contact with some people, it was not, and stuff like that can help along the way, you can unload on someone you meet often, you can be sad and worried and have concerns during the course of time.

When the nurse was able to act and give support at the right time, she utilized the time correctly and created opportunities for the patient to adapt. The nurse showed understanding for the patient's loss of time in everyday life. She supported him by saving time for the patient when possible, often through practical support in terms of planning and coordinating appointments. A long wait to start dialysis could be both good and bad as some patients became anxious while waiting, while others handled it well. All the nurses intended that the planning of care and thus the measures of support were entirely dependent on how much time they had before the patient would start dialysis. Support was therefore given priority to those patients who did not have much time to prepare. The practical assistance in various preparations for treatment was given priority over the informational and emotional support in emergency starts. The informative support deteriorated due to lack of time when the nurse had to prioritize and limit the amount of information as her experience was that the patients had a hard enough time as it was. The emotional support at emergency starts was described by one nurse:

Then again the emotional part is even more important here as they really have not been able to process anything at all. It is about saving lives then, so you have to deal with the other things later. You can notice more of an emotional reaction later as it becomes a bit of a personal crisis they go through. The others do too, but it's more apparent then.

The nurse's experience was that the emergency starts of HD are limited and unsatisfactory:

And then there are those who are started very quickly, and that is not good at all. They are not prepared for what will happen or why they are here at all … then you just have to come up with a quick solution.

### To learn about the patient with self-awareness in mind

To give support to the patient was described as to *balance an emotional journey* by the nurse. Emotional support was included in all support efforts, but the experience was that the nurse lacked knowledge of how to provide support in situations of existential suffering. It was rare that the patient showed emotions such as crying or brooding. Elderly patients, more so than younger ones, found it easier to talk about existential questions. Emotional support was harder to give to patients who did not spontaneously start to talk about their feelings. The nurse was surprised that the patients themselves made no demands for emotional support. However, she did find consolation in that the patient opened up more and more over time. The nurse gave emotional support subconsciously, which she described as: “I often think that you may not think of all the support you actually do provide, all day long … you don't always structure it, do you …?”

The nurse's experience was that the emotional support was not always received by the patient, and this depended on what phase the patient was in. The desire to provide support was there but did not always reach all the way. According to the nurse, one explanation for this could be that the patient repressed hard facts. Still, as situations arose when emotional support was given, trust made it possible to put everything else aside, fetch a cup of coffee, put a hand on the shoulder, and remain in the present. It was part of the support to try to get the patient to put their fear into words, to remain in the present themselves, and to have an open-minded attitude. The hospital chapel was a good resource but rarely used. Emotional support could be that the patient's feelings were confirmed by the nurse showing understanding, which was described as follows:

Yes, and she has also said that, I remember now, it's just this confirmation that it's okay that she feels that way. She needed to know that it was not strange to have that fear, she told me afterwards. It was a help to her that you could tell her that it is normal, you may feel this way … but she would rather get confirmation that she was not feeling well, it was quite understandable after all that she had gone through.

This category also describes the nurse's willingness and desire to improve the support to the patient during the transition to HD. This informative and practical support could be improved by the nurse evaluating the information she has and seeking new knowledge. The nurse had a positive view of the informational and practical support when she saw opportunities for improvement and found pleasure in improving patient support. There was hope for development and an openness to new ideas. The nurse described herself as a flexible, problem-solving judge of character. Experience, however, weighed heavier than evidence, mainly when it came to giving emotional support to the patient. The nurse had self-awareness and realized her lack of giving emotional support, and then chose to focus on what she knew and was familiar with, which she was good at. One nurse described: “You're hands-on … and then you do other things, that you are accustomed and familiar with, which we are really good at, informing and so on.”

Furthermore, one important thing for the nurse was to *build teamwork with the patient*, striving for the same goal together with the patient. The results showed that the nurse noticed a lack of balance in the relationship with the patient when he did not receive the support that the nurse offered. The nurse was usually happy to give support to the patient and said that she did her best. But in those situations when she felt that patients did not receive her support, she experienced feelings of inadequacy and powerlessness. Frustration arose when the support to the patient was absent because the patient didn't share his innermost thoughts and feelings. The nurse did not want to force herself on the patient, and it was then easy to take a step back or give up:

Some are difficult; you feel they put up a facade. It is not always so easy and sometimes I feel that we keep such a pleasant tone to each other and everything is fine and I feel that it's maybe not so good, and so I feel: Oh, he/she does not want to tell me more, and then I don't insist.

The nurse described how she repeatedly used her intuition when she gave support to the patient. Instead of asking the patient if they wanted to tell her more about why they were sad, the nurse changed the topic of conversation:

Yes, if there is someone who is sad and crying, I do not think it will do any good to keep talking about dialysis in that situation, but then you can always talk about their home situation, how their jobs are going.

This subcategory also describes the support given to the patient by being personally involved. A personal relationship and contact with the patient were established when the nurse took personal responsibility and made sure the patient got the support he needed. A closeness and affection developed in the relationship as the patient shared personal things, which the nurse described:

And they call about a lot of things, I think, not only about the kidney disease and their future dialysis start, but yes, they call about everything, when going through a divorce, and yes, everything really.

The nurse said that the long-lasting, personal contact was special and affected the support positively, but that there also were disadvantages to it. The nurse was aware that she would let go of the patient, but she described a desire to keep the patient with her, which showed as they kept in touch even when the patient had been transferred to HD. It was important to the nurse that the patients felt they could trust that she supported them, and therefore she could not always let go of the patients when they started in HD. An obvious risk with the long-lasting, personal contact was made clear when the personal touch pushed aside the professional support. One nurse describes this:

Then it may be one or two who find it difficult to make the decision and then, and then oh, sometimes it becomes a little bit more difficult to pressure them into it as they know me too well.

At the same time as a close personal relationship influenced the support positively, the nurse would change her support by demonstrating the patient's autonomy and making herself as superfluous as possible. This was done by encouraging active participation, teaching the patient to take responsibility for their situation, and not spoiling the patient. The patient's sense of well-being increased when the nurse gave support by confidently believing in the patient's ability and by creating an independence as she gave the power back to the patient:

For many years in healthcare it has not been like that, it is us healthcare professionals who have always known best, but we want to change that, the patient should know as much as possible himself/herself, and feel free to participate, both practically with dialysis, and also to know what makes them feel best during treatment.

### To strengthen the patient for future challenges

Support in adjusting to everyday life with dialysis treatment was given through *practice that gave the patient valuable experiences*. The patient had the opportunity to participate in the renal school, visit the dialysis unit, and meet other patients in treatment. In order to accustom the patient to the thought of dialysis treatment and to convey the theoretical knowledge through action, theory and practice were combined in the support. In renal school, the information that the nurse had provided to patients earlier was repeated so that they could obtain a complete picture of what was to come. Renal school was also relevant to the nurse as she could bring up topics and issues from it and elaborate on these when meeting with the patient. The nurse selected which patients would have the opportunity to go to renal school according to her perception of the patient's ability to embrace the information:

Some will be sent to renal school too. We hold a renal school twice a year, and we offer our patients participation. Not all, but I select the ones I think would benefit, there are usually six or seven that we offer a place each time and then maybe two to three patients come.

Visiting the dialysis clinic was done to defuse the start of dialysis and to convey a positive definition of HD. The goal was for the patient not to feel anxious and frightened before the start of dialysis, and the nurse's experience was that the patient's concern for the practical things regarding the start of dialysis decreased during the visit. The patient chose the time himself/herself, and he met both patients and staff. The nurse stayed in the background and let the patient control the conversation with a fellow patient in treatment. Patients who had dialysis were also seen as a resource for the nurse when she gave support:

There, the fellow patients may be a part of the emotional support. We don't have so many single rooms, but most patients have to share a room with someone else and you might perhaps even put a bit of thought into where you place them when they are new so they do not share a room with the most seriously ill patient.

Support to the patient during the transition to HD was affected by the nurse's cooperation with her colleagues. We were asked to *learn from others*. The nurse acted as the coordinator of the renal team, and she referred patients to colleagues when she felt that she could not provide adequate support:

Nah, in connection with the start of dialysis we convey the contact with the other categories. It should be automatic, but it's not always prior to the start but sometimes within the first few weeks. In particular regarding the diet, we can always call someone and if it's something to do with a grant we will always call the counselor.

The skills of the colleagues were used when the nurse felt the patient needed it, and she felt happy to be able to refer to colleagues in whom she had confidence. The ways of communication with colleagues were simple and quick. Using the team created satisfaction when you helped each other so that the support for the patient would be as good as possible. The support for the patient, however, could be adversely affected by the existence of problems within the organization, for instance a high workload for the dietician or difficulty in working with different booking systems. The support for the patient was occasionally unsatisfactory due to poor cooperation with the doctors. The nurse was critical of the doctors as she said they failed to cooperate and were unrealistic in relation to the patient. Different points of view and lack of communication between nurses and doctors created confusion for the patient. The consequence of this was that the nurse found it difficult to provide the support the patient needed.

And the doctor says, maybe then we wait until tomorrow, but the patient may believe that we can wait a very long time and then I, as a nurse, say we really have to be prepared for it happening very soon. Then there's a discrepancy between what we say and it's very difficult for the patient to know what really counts. I've found it difficult in that position, not to be, well, that you're not in full agreement with the doctor about what to say and that therefore, there is not a realistic expectation for the patient.

But when the partnership worked well, the nurse checked with the doctor so they would have a common picture of the patient and his needs. One nurse described the cooperation with the doctor:

Well, then, I've also in my contact with the patient talked to his attending doctor, so I've gotten feedback from him, how he/she sees it, as he may experience the patient in a different way than what I do.

It turned out that the nurse *used the closest resources* in the form of the patient's next of kin, which did not have to be a family member or a relative but could also be a good friend or caregiver. For the nurse, it was natural to involve families. Both the patient and the nurse could use the resources of the next of kin. The nurse felt that “two are better than one” when she gave informative support. It was, according to the nurse, easier for the patient to discuss matters at home if the next of kin were present at the visit to the renal clinic. The next of kin dared to ask more than the patient, so in this way the nurse could help to give support with issues she presumed would not be brought up in private conversations, which was described:

And then it's easier when a next of kin is present as there is one more that can hear and ask, and yes, there are two who can remember. Yes, I think relatives ask more, yes, they're clearly worried and as a next of kin you become highly involved, the patient is a little more exposed, so it can probably be easier for the next of kin to ask.

Next of kin became involved as the nurse pursued an open and welcoming environment at the clinic where they would feel welcome. Although it was part of the support to always involve next of kin, it was the nurse's experience that sometimes it could present difficulties. One such occasion was when she was going to give support to the patient regarding treatment options and the next of kin did not have the same opinion as the nurse. There was also a risk that it became a displaced relationship where it was felt that the next of kin was supported instead of the patient. A displaced relationship could occur when the next of kin was dominant but the patient was quiet and did not make any requests in the meeting with the nurse. An additional risk was that the next of kin became overloaded, but the nurse tried to counteract this. The nurse received confirmation of her given support when the next of kin told her the patient was satisfied:

Children may say, or family can sometimes say, they may call and have a question, and maybe then they say: Mom so often says that she feels so safe with you ….

### Comprehensive understanding

The findings of this study described how the nurse embraced the patient with threshold support for future challenges when the patient faced HD. She gave support to the patient under transition from the renal setting to hospital-bound HD as she was also very eager to gain knowledge about the patient when they first met. To get to know the patient as a special individual person was the first step and a postulate for the support, so that the patient felt confident and secure with the nurse. When she had gotten to know the patient, the informative support was given as guidance. Time was a factor that sometimes limited the support. Furthermore, the nurse's experience of giving support appeared when she received the patient aware of her own limitations in giving support. Here, she talked about her lack of tools for giving emotional support and even the frustration when the patient refused to receive her support. Finally, the nurse gave support when she used fellow patients, colleagues, and next of kin to strengthen the patient's identity in the new everyday life situation.

## Discussion

All the categories that emerged from the study explored the support during transition, but from three different perspectives. The threshold support was categorized in “the essential part of gaining knowledge about the patient” and explored the support beginning at the meeting with patients at the renal setting. The next category was “to learn about the patient with self-awareness in mind,” which explored the support based on the nurse's self-awareness and balance in the patient relationship. The third category was “to strengthen the patient for future challenges” and explored the support based on the nurse's use of fellow patients, colleagues, and next of kin as she provided support.

The nurse's experience in providing support to the patient during the transition to HD implied a threshold support. Both informative and practical support was structured and was consciously given as it was carefully planned and followed up. Informative, practical, and emotional support was intertwined and given with interest in the patient as an individual.

The emotional support was distinctive compared to the informative and practical support. It was sometimes subconscious and not so structured, and it could be given at any time during the transition. The nurse's experience was that it was difficult and that she lacked knowledge about providing emotional support.

The actual transition may be described as a constant movement through time and space, extending from the first visit to the renal nurse to the patient's first dialysis treatment. Hutchinson (2005) argues that the patient during this time is in need of assistance during several transitions. In life, several transitions, self-selected or not, may coincide (Chick & Meleis, 2007; Schumacher & Meleis, 1994). These can be anything from support when informed of the diagnosis of CKD, at the loss of time and freedom, or at the construction of access to the final support at the start of dialysis (Hutchinson, 2005). The transitions can be compared to thresholds that patients must overcome. The term “threshold support” is taken from the literary concept of the chronotope. The concept of chronotope (time-space) is defined as a combination of spatial and temporal characteristics of a meaningful and concrete whole (Brandorf, 2009). A connection between transition and chronotope can be made, as Chick and Meleis (2007) and even Schumacher and Meleis (1994) argue that it is important that the experience of the transition makes sense. In his study, where he highlights Hjalmar Bergman's novels, Brandorf (2009) addresses the threshold as a local chronotope. The threshold is a point, a borderline position, where the crisis, change, or alternation between different states takes place. The process of crisis is often located in threshold rooms, rooms that reveal or indicate a crisis-like transformation. A parallel can be drawn to the clinic room where the support during the transition is formed. When the patient has been diagnosed with CKD and the transition to HD takes place, life becomes significantly more restricted (Molzahn et al., 2008). The patient is in a crisis situation where he is undergoing a transformation of his identity. A struggle takes place to come to terms with a new world where the relationship with oneself, others, health, and treatment has changed in a fundamental way (Hutchinson, 2005). The choice of the term “threshold support” in this study has its origin in this comparison between transition and chronotope.

### Learn from the patient's standpoint

In order to provide support to the patient, the nurse began to develop a relationship of trust with the patient. The basis for this was that they got to know each other in conversation where informational support, practical support, and also emotional support were intertwined. The informational support was well structured and was given considering the patient's individual needs, and it included planning, continuity, anticipation, and feedback. The nurse pointed out the importance of a genuine interest in the patient's everyday life. By forming an opinion on where the patient was in life, the nurse built up trust between herself and the patient. Ballerini and Paris (2006) confirm the importance of the nurse's interest in the patient's entire life situation in their study by describing today's patients as more enlightened and aware of their rights. They are not satisfied with just the test results and the latest medical care. The patient expects to meet an empathizing nurse, who listens and understands the difficulties in adapting to the new everyday life. Today's patients ask for consideration for the personal quality of everyday life (Ballerini & Paris, 2006). In the results of the study, the nurse did not herself point out any significant deficiencies in the conversation with the patient regarding the informational support; she appeared, rather surprisingly, confident in her informative role. Nevertheless, it was clear that the nurse was not satisfied with the patient's acceptance of her informative support. On several occasions, the nurse reiterated that she “read” the patient during the course of the conversation, which can be a good nursing task in itself, as long as it does not take priority over evidence-based knowledge. None of the nurses in the study claimed to have training in conversational techniques, crisis management, or the like.

According to Berg et al. (2008), experience-based knowledge is a key part of evidence-based care. Research, preferably with different epistemological positions, should be an addition to the nurse's experience-based knowledge (Berg et al., 2008). When the nurse provides support, it is about her having as good a foundation of knowledge as possible. A good foundation of knowledge cannot consist only of a nurse's experience. The different sources of a good foundation of knowledge can, however, be research, patient experience, clinical experience, and local information (Scott & McSherry, 2008). The informational support of the patient includes knowledge of not only what to inform about but also how we should talk in order to bring out the patient's knowledge, opinion, and experience. As Seeberger (2010) describes it, the nurse must have the patient with her in the conversation.

Support to patients with CKD also meant guiding the patient in choosing a treatment option. The information was intended to provide insight into the different treatment options, with the exception of transplantation when this treatment was not feasible. Difficulty in providing support in the form of guidance in treatment options could occur when the nurse knew what the most medically appropriate treatment was, but the patient did not agree. Even though she found it difficult in these situations, the nurse had the ability to see to the patient's desire instead of the medically correct choice. Here, she worked according to Polaschek's (2003) negotiated care model. Simplified, this model means that the effort of the nurse lies in listening and in taking into account the patient's experience. The nurse should not only perform specific clinical tasks associated with dialysis, or only implement what is the best option for the patient from a professional point of view. She should also try to adapt to what the best options are from the patient's point of view, in order to help the patient master a somewhat normal everyday life (Polaschek, 2003). The nurse described her role in the encounter with the patient as giving support by providing hope in hopelessness and giving hope of freedom in restriction. In line with this, it has been shown that some patients during the transition to HD make a positive reevaluation of their lives. To feel hope and optimism for the future is important and helps them manage the transition (Mitchell et al., 2009).

The result showed that the nurse's support for the patient was adapted depending on the time she had available. The situation was considered optimal when the nurse had the time she needed to prepare the transition in the best possible way, and the overall experience was that the patient needed plenty of time to accept his/her situation. When there was enough time, the nurse's experience was that the patient experienced the transition with less anxiety. In emergency starts, which according to the nurses happened too often, the emotional support was not given priority and instead focus was given to the medical issues. Research shows that early referral to the renal setting is crucial for patient survival in HD. The risk of cardiovascular disease and a premature death prior to an HD start is also reduced early referrals (Crowley, 2003; Sijpkens, Berkhout-Byrne, & Rabelink, 2008). Timing is also important for the emotional support as the development of the patient's disease is emotionally traumatic and often causes both total denial and anger in the patient. Therefore, it is essential that there is adequate time for the renal team to assist the patient in the emotional preparation for the inevitable lifestyle changes that occur (Crowley, 2003). Early education allows patients to learn about their illness in peace and quiet, and they may then find it easier to adapt psychologically to the transition. It is thus important for the patient to have consistent access to the renal team during the transition to HD in order to receive guidance, emotional support, and guidelines through his/her health crisis (Neyhart, McCoy, Rodegast, Gilet, Roberts, & Downes, 2010).

### Exchange knowledge

Emotional support lacked a structural equivalent to informational support. The nurse had no knowledge of how to provide emotional support, especially regarding the existential concerns of the patient. Although the nurse described herself as a “flexible, problem-solving: judge of character,” she lacked the ability to get the patient to open up. When the patient did not tell the nurse about his/her needs, the consequence could be that the emotional support was left out. The nurse was surprised that the patient did not make any major demands for emotional support, but found some consolation in that it became easier for the patient to open up as time passed. Herlin and Wann-Hansson (2010) showed that patients who had started in HD felt dependent on the nurse. They expressed a need to be able to trust her, noting that some nurses understand their patient's fear of dying, while some do not understand it at all. A similar situation might occur at the renal setting, and then it would be the nurse's task to make the first move and get the patient to open up. But instead of being self-critical, the nurse referred to the patient as repressing cold facts, being difficult and reluctant to talk about his/her need for emotional support. Davison and Jhangri (2010) suggest that patients with CKD are often in need of emotional support, especially when they seek meaning and hope in their situation. Patients who cannot deal with their existential worries endure unnecessary suffering. More than half of the patients with CKD feel it is essential that their existential concerns are taken seriously by the staff, and they expect to receive emotional support from the renal nurse (Davison & Jhangri, 2010). In relation to the growing trend where nurses are required to perform more and more patient-centered nursing care, you cannot ignore the fact that the patient has the right to emotional support when the need exists.

The study's findings include that the nurse is aware of the need for emotional support, and when the patient finally asked for emotional support, it was the nurse's experience that she could provide the support the patient needed. According to the nurse, the problem was the meetings, during which the nurse could not reach the patient. The nurse's experience was that the informational support, at which she considered herself an expert, was not received by all patients in an optimal way. This situation could be explained by the patient's lack of emotional support. Antonovsky (2005) describes this when he studies the way people deal with the difficulties that we encounter in life. Patients came to the nurse with varying ability to express their feelings, which can be related to the concept of “sense of coherence” (Antonovsky, 2005). Some patients had a strong sense of coherence, which could be related to them understanding what was happening, having the ability to handle the experience, and feeling that life was worth the effort. The nurse's experience was that it was easier to give support to the patients who had this basic trust. The challenge was to support those with a low sense of coherence: that is, those patients who could not convey to the nurse when they did not understand the information, who could not find a meaning in the situation, and who did not know how to handle it. One risk was that these patients could not process the transition because the nurse, as she herself puts it, “did not want to force herself on them.” According to Klang et al. (1999), patients are responsive and active once they start with HD if they get the emotional support they need. There is an important balance for the nurse to strengthen the autonomy of the patient, but at the same time not leaving them alone with too hard decisions to make (Delmar, Alenius-Karlsson, & Höjer-Mikkelsen, 2011).

The nurse is able to refer patients to a counselor in the renal team when there is a need for it. The results showed that the nurse did not use this resource as often as she could. Davison and Jhangri (2010) describe in a study that physicians who feel uncomfortable because they lack the skill to manage the patient's existential concerns still need the ability to discover and respect the unfulfilled existential need that is causing considerable concern for the patient. It is also important to understand the impact that raw existential concerns may have on the patient's clinical picture. In this situation, the patient should be referred to other healthcare providers who have knowledge of how to help the patient deal with existential concerns (Davison & Jhangri, 2010).

### Strengths and limitations of the study

This study is limited to the stories of eight participating nurses in the south of Sweden. However, it might encourage both researchers and nurses as to how nurses prepare patients during the transition to HD treatment. The strength of the study was to investigate the nurses’ perspective of giving support to patients in transition to HD treatment.

## Conclusion

The consistent theme was “Prepare the patient with threshold support for future challenges when facing hemodialysis.” The results showed that the nurse described that she gave support with openness and awareness of the patient's personal needs by following the three main categories: the essential part of gaining knowledge about the patient, to learn about the patient with self-awareness in mind, and to strengthen the patient for future challenges. All categories included informative, emotional, and practical support and were given with an interest in the individual and their everyday life, though the experience was that the nurse also subconsciously gave support to the patient. However, the nurse's emotional support to patients had flaws as she lacked the knowledge of how she could reach the patient and get him to talk about his/her experiences of the stressful transition. Because of this, she could not give to all patients the individual support they needed to manage the transition.

To prepare the patient for the future, the nurse must be aware of the importance of the emotional support to the patient and their sense of coherence. Nurses can increase their self-awareness by actively seeking new scientific knowledge. Encouragement and, above all, being given the time by the ward manager are musts to make this a reality. The Swedish Renal Medical Society of Nursing may also contribute to increased knowledge by organizing educational days on the subject. As the nurse needs to ask the patient more difficult questions, she needs training in conversational techniques. The most important educational intervention needed is the already sought-after specialized training for renal nurses, and this needs to happen imminently. Knowledge and skill in providing emotional support should have a prominent role in this education, which is equivalent to other kinds of specialist training for nurses. In addition to education, when it comes to providing emotional support, nurses can take advantage of the competence of other care providers a lot more than what they do today. A more transparent collaboration between pastoral care, counselors, and nurses in view of the patient's individual needs would therefore be desirable. Conveying counselor contact should be as urgent whether you need emotional support or support with financial matters. Through awareness and the correct knowledge, nurses not only can provide informative and practical support professionally; with the emotional support, they can also guide the patient to become a confident, involved, and responsible individual.

Further research into the nurse's emotional support for patients is needed from both the patient's and the nurse's perspective. Such research could form the basis for the development of measuring instruments with which the nurse can monitor the patient's sense of coherence. It would also be interesting to see if patients find it easier to communicate with nurses about their emotional needs through virtual means of communication rather than normal conversations.
